# Special Issue of *International Journal of Molecular Sciences* (*IJMS*) “Purinergic P2 Receptors: Structure and Function”

**DOI:** 10.3390/ijms22010383

**Published:** 2020-12-31

**Authors:** Hana Zemkova

**Affiliations:** Institute of Physiology, Czech Academy of Sciences, 14220 Prague, Czech Republic; Hana.Zemkova@fgu.cas.cz

This Special Issue of *International Journal of Molecular Sciences* (*IJMS*) contains 7 reviews and 12 original research papers written by a panel of experts who highlight recent advances in molecular structure and cellular function of purinergic P2 receptors.

## 1. Research

The original articles cover a range of topics from structure–function relationships to physiology and pharmacology of P2X and P2Y receptors. The P2X4 receptor is well known to be allosterically modulated by Zn^2+^ binding to C132 and C149 residues, forming disulfide bond SS3 in the head receptor domain. Here, Peralta et al. examined the importance of acid residues E160 and D170 in the SS2/SS3 microenvironment for sensitivity to zinc. The authors showed that both residues play a role in the positive allosteric modulation of P2X4 receptor as putative Zn^2+^ ligands [1]. Popova et al. deeply explored the complexity of P2X4 receptor modulation by ethanol. Using site-directed mutagenesis, two electrode voltage-clamp electrophysiology in *Xenopus* oocytes, and molecular models of P2X4 receptor in both closed and open states, the authors showed that transmembrane residues R33 in the TM1 segment and residue D354 of the neighboring TM2 segment interact and contribute to channel function and ethanol sensitivity [2]. Rupert et al. performed alanine mutagenesis of conserved and/or receptor-specific residues in the extracellular vestibule of rat P2X7 to investigate the role of these residues in receptor function. The authors found that clusters of conserved residues proximal to transmembrane domain are important for receptor structure, membrane expression, and/or channel gating, and receptor function is critically dependent on the properties of the nonconserved residue F322, at the top of the extracellular vestibule and between the extracellular and central vestibules, which stabilizes the closed state of the P2X7 receptor channel [3]. The study conducted by Bautista-Perez et al. strengthens the notion that P2X7 receptors play a central role in the renal inflammation associated with angiotensin II-induced hypertension. The authors found that pharmacological inhibition of P2X7 receptors improves glomerular hemodynamics alterations resulting from angiotensin II infusion, and reduces immune cell infiltration and expression of proinflammatory cytokines and NLP3 [4]. Wilkaniec et al. present data showing that activation of P2X7 by alpha synuclein in neuroblastoma SH-SY5Y cells is responsible for reactive oxygen species (ROS)-mediated mitochondrial dysfunction as well as deregulation of AMP-activated protein kinase AMPK and parkin that might result in the accumulation of defective mitochondria [5]. Miteva et al. investigated interaction between intracellular calcium chelators and the activity of P2X7 receptors in mouse neuromuscular junction. This study shows that the activation of P2X7 receptors in motor nerve endings may provide additional Ca^2+^ entry into motor nerve terminals, which, independent of the modulation of L-type voltage-dependent calcium channel activity, can provide sufficient Ca^2+^ signals for ACh secretion [6]. Koldej et al. found that there is an association between P2X7 polymorphisms and post-transplant outcomes in allogeneic hematopoietic stem cell transplantation, which represents a highly effective treatment method for hematologic malignancies. The authors found that variations in recipients or donors may have a minor but clinically nominal impact on transplantation outcomes. For example, the loss-of-function mutant G496A is overrepresented in recipients who did not develop severe acute graft versus host disease and is associated with improved overall survival in rare homozygous recipients [7]. The study by Zhang et al. revealed that some P2Y receptor subtypes are differently regulated during human mesenchymal stem cell differentiation towards endothelial cell and smooth muscle cell lineages—P2Y4 and P2Y14 are involved in the early stage commitment, while P2Y1 is the key player in controlling mesenchymal stem cell differentiation towards either endothelial or smooth muscle cells [8]. Christensen et al. show that inhibition of P2Y1 by constant infusion of specific inhibitor MRS2500 markedly increased the survival in mice with induced sepsis, partially prevented the sepsis-induced depletion of circulating thrombocytes, and dampened the sepsis-associated increase in proinflammatory cytokines. In contrast, the P2Y12 receptor inhibition had only a marginal effect in vivo and in vitro [9]. It is known that hepatocytes from CCl_4_-treated fibrotic mice exhibit increased P2Y2-mediated purinergic responses that are involved in the onset of the fibrotic damage associated with the reversible phase of the hepatic damage promoted by CCl_4_. Velazquez-Miranda et al. performed hepatic transcriptional analysis by microarrays upon CCl_4_ administration, showing that P2Y2 activation regulates diverse pathways, revealing complex action mechanisms [10]. The purinergic receptor P2Y14 is the only P2 receptor that is stimulated by uridine diphosphate UDP. Mikolajewicz et al. examined the role of P2X14 in bone formation by using murine osteoblasts, the P2Y14 inhibitor 4,7-disubstituted 2-naphthoic acid derivative (PPTN), or by generating CRISPR-Cas9-mediated P2Y14 knockout C2-Ob clones (Y14_KO_). This study revealed that P2Y14 reduces cell responsiveness to mechanical stimulation and mechanotransductive signaling, and modulates osteoblast differentiation [11]. Extracellular ATP or UTP also activates plasma membrane-targeted TMEM16A protein, which operates as a Ca^2+^-activated Cl^−^channel in secretory epithelial cells of the exocrine pancreas and salivary glands. Schreiber et al. found that this effect occurs through stimulation of purinergic P2Y receptors and increase in intracellular Ca^2+^ concentration by a Ca^2+^ release from the endoplasmic reticulum [12].

## 2. Review

Review articles present several up-to-date aspects of the biology of P2X and P2Y receptors that have both, independently or together, been implicated in the physiology or pathophysiology of endothelial, cardiovascular, immune, circadian, neuromuscular, and nervous system. Strassheim et al. reviewed data showing that P2Y receptors have been established as a potential target for endothelial dysfunction and cardiovascular diseases; moreover, they discussed a role of these receptors in hypertension, vascular tone, aging, diabetes, oxidative stress, atherosclerosis, vascular inflammation, metabolic syndrome, vascular permeability, and neovascularization [13]. Wirsching et al. made an insightful review of how extracellular ATP and expression of P2X and P2Y receptors within the cells of the distal lung contribute to alveolar function, surfactant secretion, epithelial fluid transport, host defense, and the development/progression of lung diseases such as infectious pneumonia and acute respiratory distress syndrome [14]. Ali et al. summarized recent data about P2X and P2Y receptor expression and function within the suprachiasmatic nucleus, retina, and peripheral circadian oscillators exemplified by urinary bladder, suggesting that interaction between the circadian system and purinergic signaling might play a role in rhythmic body functions under physiological and pathological conditions [15]. Among the P2X receptors, P2X7 has attracted increasing interest in the field of inflammation and/or immune response as well as in cancer. Rivas-Yanez et al. reviewed the role of P2X7 in maturation and migration of antigen-presenting dendritic cells, activation and migration of naïve T cells, inflammasome activation, cytokine secretion, and apoptosis induction [16]. Panicucci et al. explored the interactions between the immune system and muscle regeneration. This review summarizes recent knowledge about ATP-P2X7 signaling, ATP release, metabolism, feedback control, cross-talk of P2X7 receptor with members of muscle inflammasome, and regulation of P2X7 signaling by pannexins and connexins, all of which are explored in the context of muscular dystrophies [17]. Another review by Martinez-Cuesta et al. focuses on the structure–function relationships of P2X7 receptors, polymorphism and transcriptional regulation of P2X7, extracellular ATP levels that are critical to the full activation of the P2X7 receptor, cell localization of this receptor, interactions with intracellular signaling pathways, and allosteric modulation by endogenous molecules in different species and cell types [18]. Lastly, even if no specific receptor has been cloned for GTP or its metabolites, specific binding sites for GTP have been found in nervous tissue and muscle cells. Mancinelli et al. discuss a possible role of adenine- and guanine-based nucleotides during embryogenesis and development, investigate possible mechanisms for the extracellular pool formation, and explore specific effects of guanine-based nucleotides in excitable tissues [19].

This Special Issue underlines the richness but also the complexity of P2Y and P2X receptors in cardiovascular, immune, circadian, and nervous systems. The present challenge is to accelerate the exploration of these receptors as unique pharmacological targets for clinical applications.

## References

[1] Peralta F.A., Huidobro-Toro J.P. (2020). New Insights of the Zn(II)-Induced P2X4R Positive Allosteric Modulation: Role of Head Receptor Domain SS2/SS3, E160 and D170. Int. J. Mol. Sci..

[2] Popova M., Rodriguez L., Trudell J.R., Nguyen S., Bloomfield M., Davies D.L., Asatryan L. (2020). Residues in Transmembrane Segments of the P2X4 Receptor Contribute to Channel Function and Ethanol Sensitivity. Int. J. Mol. Sci..

[3] Rupert M., Bhattacharya A., Stillerova V.T., Jindrichova M., Mokdad A., Boué-Grabot E., Zemkova H. (2020). Role of Conserved Residues and F322 in the Extracellular Vestibule of the Rat P2X7 Receptor in Its Expression, Function and Dye Uptake Ability. Int. J. Mol. Sci..

[4] Bautista-Pérez R., Pérez-Méndez O., Agustina C.-M., Pacheco U., Santamaría J., Rodríguez-Sámano F., Rodriguez-Iturbe B., Navar L.G., Franco M. (2020). The Role of P2X7 Purinergic Receptors in the Renal Inflammation Associated with Angiotensin II-Induced Hypertension. Int. J. Mol. Sci..

[5] Wilkaniec A., Cieslik M., Murawska E., Babiec L., Gassowska-Dobrowolska M., Palasz E., Jesko H., Adamczyk A. (2020). P2X7 Receptor Is Involved in Mitochondrial Dysfunction Induced by Extracellular Alpha Synuclein in Neuroblastoma SH-SY5Y Cells. Int. J. Mol. Sci..

[6] Miteva A., Gaydukov A., Balezina O. (2020). Interaction between Calcium Chelators and the Activity of P2X7 Receptors in Mouse Motor Synapses. Int. J. Mol. Sci..

[7] Koldej R.M., Perera T., Collins J., Ritchie D.S. (2020). Association between P2X7 Polymorphisms and Post-Transplant Outcomes in Allogeneic Haematopoietic Stem Cell Transplantation. Int. J. Mol. Sci..

[8] Zhang Y., Babczyk P., Pansky A., Kassack M.U., Tobiasch E. (2020). P2 Receptors Influence hMSCs Differentiation Towards Endothelial Cell and Smooth Muscle Cell Lineages. Int. J. Mol. Sci..

[9] Christensen M.G., Johnsen N., Skals M., Hamilton A.D.M., Rubak P., Hvas A.M., Praetorius H. (2020). Prevention of P2 Receptor-Dependent Thrombocyte Activation by Pore-Forming Bacterial Toxins Improves Outcome in A Murine Model of Urosepsis. Int. J. Mol. Sci..

[10] Velazquez-Miranda E., Molina-Aguilar C., Gonzalez-Gallardo A., Vazquez-Martinez O., Diaz-Munoz M., Vazquez-Cuevas F.G. (2020). Increased Purinergic Responses Dependent on P2Y2 Receptors in Hepatocytes from CCl4-Treated Fibrotic Mice. Int. J. Mol. Sci..

[11] Mikolajewicz N., Komarova S.V. (2020). Role of UDP-Sugar Receptor P2Y14 in Murine Osteoblasts. Int. J. Mol. Sci..

[12] Schreiber R., Ousingsawat J., Kunzelmann K. (2020). Targeting of Intracellular TMEM16 Proteins to the Plasma Membrane and Activation by Purinergic Signaling. Int. J. Mol. Sci..

[13] Strassheim D., Verin A., Batori R., Nijmeh H., Burns N., Kovacs-Kasa A., Umapathy N.S., Kotamarthi J., Gokhale Y.S., Karoor V. (2020). P2Y Purinergic Receptors, Endothelial Dysfunction, and Cardiovascular Diseases. Int. J. Mol. Sci..

[14] Wirsching E., Fauler M., Fois G., Frick M. (2020). P2 Purinergic Signaling in the Distal Lung in Health and Disease. Int. J. Mol. Sci..

[15] Ali A.A.H., Avakian G.A., Von Gall C. (2020). The Role of Purinergic Receptors in the Circadian System. Int. J. Mol. Sci..

[16] Rivas-Yáñez E., Barrera-Avalos C., Parra-Tello B., Briceño P., Rosemblatt M.V., Saavedra-Almarza J., Rosemblatt M., Acuña-Castillo C., Bono M.R., Sauma D. (2020). P2X7 Receptor at the Crossroads of T Cell Fate. Int. J. Mol. Sci..

[17] Panicucci C., Raffaghello L., Bruzzone S., Baratto S., Principi E., Minetti C., Gazzerro E., Bruno C. (2020). eATP/P2X7R Axis: An Orchestrated Pathway Triggering Inflammasome Activation in Muscle Diseases. Int. J. Mol. Sci..

[18] Martinez-Cuesta M.A., Blanch-Ruiz M.A., Ortega-Luna R., Sanchez-Lopez A., Alvarez A. (2020). Structural and Functional Basis for Understanding the Biological Significance of P2X7 Receptor. Int. J. Mol. Sci..

[19] Mancinelli R., Fanò-Illic G., Pietrangelo T., Fulle S. (2020). Guanosine-Based Nucleotides, the Sons of a Lesser God in the Purinergic Signal Scenario of Excitable Tissues. Int. J. Mol. Sci..

